# Supplementary material for: Antibiotic Treatment Induces Long-Lasting Effects on Gut Microbiota and the Enteric Nervous System in Mice
Source: Antibiotics (Basel). 2023 Jun 1;12(6):1000. doi: 10.3390/antibiotics12061000 (PMC10295661; doi:10.3390/antibiotics12061000)
Supplement: Supplementary file 1 [file antibiotics-12-01000-s001.zip › antibiotics-2394376-supplementary.pdf]

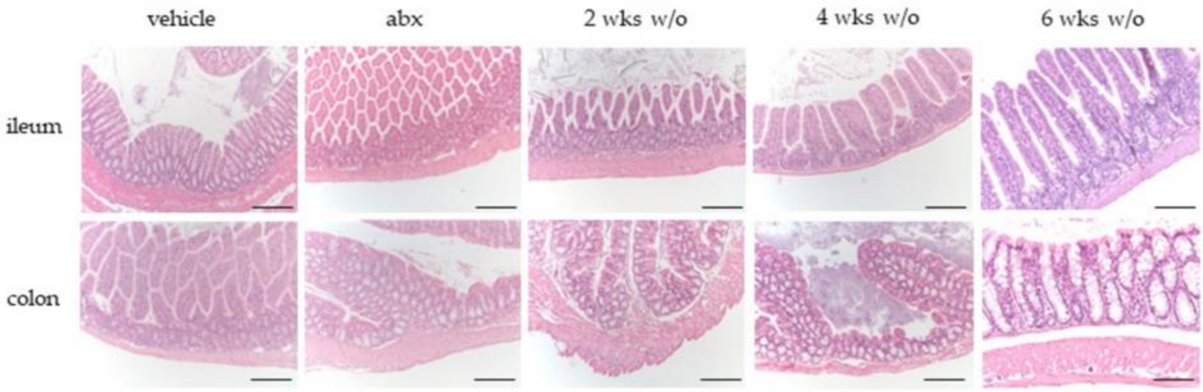

Figure S1. Histological analysis of the ileum and colon. Representative cross-sections (number of mice for each experimental group = 4 mice per group) stained with H&E of vehicle-treated, antibiotic (abx) treated mice and abx treated mice sacrificed following 2, 4, and 6 weeks (wks) post-antibiotic wash-out (w/o). Original magnification 40X. Scale bar, 150 μm.
